# Next Generation CD40 Agonistic Antibodies for Cancer Immunotherapy

**DOI:** 10.3389/fimmu.2022.940674

**Published:** 2022-07-13

**Authors:** Ran Salomon, Rony Dahan

**Affiliations:** Department of Systems Immunology, Weizmann Institute of Science, Rehovot, Israel

**Keywords:** CD40, fc receptor, agonistic antibody, therapeutic antibody, cancer immunotherapy, bispecific antibodies (BsAbs)

## Abstract

The clinical use of anti-CD40 agonist monoclonal antibodies (mAbs) is aimed at recruiting the immune system to fight the tumor cells. This approach has been demonstrated to be effective in various preclinical models. However, human CD40 Abs displayed only modest antitumor activity in cancer patients, characterized by low efficacy and dose-limiting toxicity. While recent studies highlight the importance of engineering the Fc region of human CD40 mAbs to optimize their agonistic potency, toxicity remains the main limiting factor, restricting clinical application to suboptimal doses. Here, we discuss the current challenges in realizing the full potential of CD40 mAbs in clinical practice, and describe novel approaches designed to circumvent the systemic toxicity associated with CD40 agonism.

## Introduction

The field of immuno-oncology has progressed steadily over the last decade. Immunotherapy has joined the ranks of surgery, chemotherapy, radiation, and targeted therapy in the arsenal of cancer treatments (1, 2). An increasing number of immune checkpoint-targeted monoclonal antibodies (mAbs) have been developed with the aim of harnessing the immune system to eradicate tumor cells (3, 4). These efforts have resulted in successful clinical application of blocking mAbs against CTLA-4 and PD-1/PD-L1 checkpoints on T lymphocytes (T cells) to induce effective tumor-eliminating immunity. However, a remaining unmet clinical challenge is to stimulate immunity against “cold” tumors, which lack significant immune infiltration at treatment onset. Agonist mAbs targeting the cluster of differentiation 40 (CD40) immune receptor emerge as a potential approach to increase the number and quality of tumor-infiltrating T cells (TILs) and, thereby, response effectiveness, either as a monotherapy or to reverse resistance to checkpoint-blocking antibodies (5–9).

CD40 is a tumor necrosis factor receptor (TNFR) superfamily member. It is expressed on antigen-presenting cells (APCs) including dendritic cells (DCs), B cells, macrophages, classical and non-classical monocytes (10–12), on a variety of non-immune cells including platelets and endothelial cells (13, 14), and on several types of tumor cells (15). CD40 plays a central role in stimulating immune synapses, including during T cell priming by APCs, when its interaction with the CD40 ligand (CD40L) licenses DCs to activate antigen-specific T cells (5, 16). This is accomplished through the upregulation of major histocompatibility complex (MHC) molecules, increased expression of the costimulatory molecules CD86/CD80, and upregulation of TNF superfamily ligands on the DC surface, as well as by secretion of interleukin-12 (IL-12), which fuels CD8^+^ T cell activation. Likewise, the CD40/CD40L axis plays a central role in the B-T cell immune synapse, promoting B cell activation and proliferation as well as antigen presentation (5, 6, 11, 16).

Agonistic anti-CD40 Abs are designed to mimic CD40L by crosslinking CD40 and, thereby, promote the maturation of DCs and improve their antigen presentation capabilities. This results in expansion of tumor antigen-specific cytotoxic T cells, which can lead to the eradication of tumors (5, 17, 18). Motivated by promising results in a variety of cancer animal models, several human CD40 mAbs have been developed and evaluated in clinical trials over the last two decades (6, 19–22). However, the preclinical potency has not yet been recapitulated in clinical setting and none of these mAbs has advanced beyond early trial phases. Among the challenges that were encountered during these evaluations are low detected levels of immune activation and high toxicity levels associated with the treatment. The toxicity limited the use of CD40 mAbs to suboptimal doses, resulting in insufficient immune activation and antitumor efficacy (21, 23–26). Here, we highlight key factors and cellular pathways associated with effective agonism and the observed clinical toxicity. Furthermore, we describe recent antibody-engineering approaches and treatment regimens that we find the most advanced and promising in the quest to overcome the challenges preventing the clinical use of CD40 agonistic mAbs.

## Harnessing FcγRs to Potentiate the Activity of CD40 mAbs

**Figure 1:**
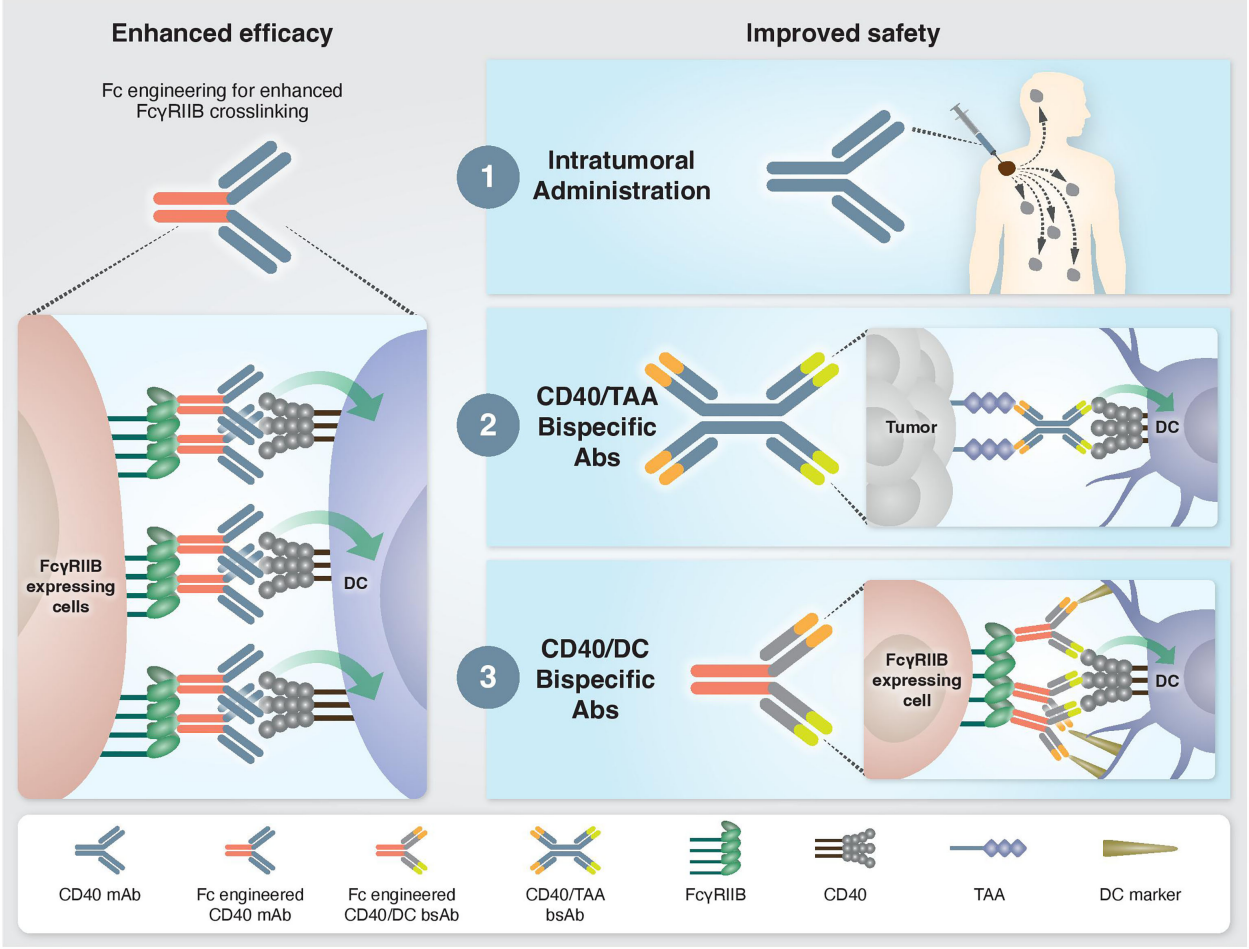
Approaches to enhance the efficacy and safety of CD40 agonistic mAbs. Left: Enhanced CD40 agonism by Fc-engineered mAbs designed to increase FcγRIIB-mediated crosslinking. Right: Approaches to bypass treatment associated toxicities. 1) Intratumoral administration. Injection of low mAb dose directly into the tumor enables local antitumor immune activation without systemic side effects. 2) Tumor-targeted bispecific CD40 antibodies direct the agonistic antibody to the TME by targeting tumor-associated antigens, which are overexpressed and/or selectively expressed at the tumor site. 3) Dendritic cell-targeted bispecific CD40 antibodies direct the agonistic antibody to the cell types that drive treatment-associated antitumor activity but not toxicity.

Fc-gamma receptors (FcγR) are central players in the *in vivo* agonistic activity of CD40 mAbs (25, 27–29). This Fc-mediated mechanism involves higher order crosslinking of the CD40 mAbs by FcγRIIB expressed in trans by cells neighboring the CD40-expressing cells. This results in enhanced clustering of CD40 on the target cell and, consequently, increased CD40 signaling. The relatively low clinical response elicited by different anti-human CD40 mAbs (15, 19) can be attribute to the structure of their IgG scaffold, which is not optimized for FcγRIIB binding. It was demonstrated that the *in vivo* activity of human CD40 mAbs is dependent on their affinity to FcγRIIB and, notably, this activity was significantly improved by Fc engineering (25) (**Figure 1**). Following this preclinical observation, a second generation of Fc-engineered anti-human CD40 mAbs with enhanced FcγRIIB binding are now being tested in clinical trials (25, 30–32).

One such antibody is 2141-V11. Based on selicrelumab, the original IgG2 isotype was converted into IgG1 and the affinity to FcγRIIB was selectively enhance by Fc engineering. The Fc-engineered version of this mAb displayed a significantly enhanced *in vivo* antitumor response compared to the parental IgG2 variant in multiple tumor models, includes melanoma, colon adenocarcinoma and bladder cancer (25, 30, 31). APX005M (sotigalimab) is another CD40 mAb that was Fc-engineered to increase the interaction with FcγRIIB, now evaluated in several early-phase studies (32, 33). Different Fc mutations were introduced to the IgG1-Fc scaffold of 2141-V11 and APX005M. While the binding of 2141-V11 is enhanced selectively to FcγRIIB and not to other FcγRs, APX005M engages both the inhibitory FcγRIIB and the activating FcγRIIA^131R^. Preclinical studies showed increased *in vivo* agonistic activity for both Fc-engineered mAbs over their parental non-mutated IgG1 variant (25). However, due to the opposite effect of decreased mAb potency upon engagement with FcγRIIA, the FcγRIIB-selective enhanced Fc variant displayed superior agonistic activity.

Because crosslinking of CD40 on the membrane surface is key for the activity of CD40 agonists, various strategies to enhance CD40 receptor trimerization have been developed. These Fc-independent approaches include hinge engineering to the unique structural configuration of IgG2 subclass, which was reported to enhance CD40 agonistic activity. Mutations of specific cysteines in CD40 agonistic mAbs are used to prevent shuffling of disulfide bonds between the IgG2 hinge and CH1 regions, thus locking the hinge conformation that contributes to enhanced CD40 clustering (34, 35). Other approaches to promote CD40 receptor multimerization include the use of recombinant CD40L-based instead of antibody-based molecules (36), or utilizing Fc-docking scaffolds to multimerize anti-CD40 mAbs (37). A notable difference between these Fc-dependent and Fc-independent engineering approaches is the requirement for FcγRs engagement in addition to CD40 engagement in the former but not the latter, which may results in different biodistributions of these molecules. The consequences of these distinct properties for the mechanism and therapeutic index of these reagents should be clarified in future studies. A combination of different approaches to enhance agonism was also suggested in a study demonstrating synergistic agonist potency of a combined hinge and Fc-engineering strategy (38).

While these Fc and protein engineering strategies can improve the antitumor efficacy of CD40 agonists, the stronger potency of these next-generation agonists is accompanied by an increase in side effects and toxicity that characterize this type of immunotherapy (25, 30). Consequently, the systemic administration of these agonists is limited to suboptimal doses and their full potential could not be exploited.

## Side Effects and Toxicities of CD40 mAbs

As mentioned, human CD40 agonistic mAbs were reported to trigger severe adverse effects and toxicities. These include hepatotoxicity, cytokine release syndrome (CRS) (19, 20, 39), thrombocytopenia (19, 24, 25, 30), general hyperimmune stimulation (40), and tumor angiogenesis in response to endothelial cell activation (41). The broad expression of CD40 by various immune and non-immune cells types in the tumor and in other organs is likely to contribute to the occurrence of these side effects.

Recent studies highlighted the role of macrophages, Kupffer cells, platelets and neutrophils in mediating liver toxicity. Using a single-cell RNA sequencing approach, Siwicki et al. described a mechanistic interplay involving IFN-γ-secreting lymphocytes and IL-12-producing tissue-resident Kupffer cells, resulting in liver toxicity (42). This anti-CD40 mediated hepatotoxicity is associated with an IL-12-dependent accumulation of MHC II^+^, CD14^+^ and CD11b^+^ macrophages in the liver (43). It was further shown that IL-12 and IFN-γ were not toxic by themselves and that neutrophils respond to these two cytokines by upregulation and secretion of TNF, the levels of which determine the severity of liver toxicity. Another player in the network that mediates the toxic effect of CD40 mAbs on the liver are platelets. In a recent study, we have demonstrated the causal role of macrophages and platelets in liver toxicity after CD40 treatment (44). Systemic cell depletion of macrophages or platelets completely abrogated the elevation in liver transaminases (ALT/AST) that was observed after anti-human CD40 treatment. While these findings highlight the involvement of macrophages, Kupffer cells, platelets and neutrophils, the full mechanistic interplay between these players driving liver toxicity following anti-CD40 treatment still needs to be elucidated.

In the clinic, CRS was evident within minutes to hours after CD40 mAb infusion and was associated in these patients with elevation in serum IL-6 (19). *In vivo* upregulation of intracellular IL-6 was detected by classical CD11c^-^ monocytes in the blood, lymph nodes and spleen, after immunization with anti-human CD40 mAb in humanized CD40 mouse strain (44). This suggests monocytes as the major cell population driving IL-6 secretion.

DCs and, in particular, the conventional type-1 dendritic cells (cDC1s) are essential for CD40-targeted immunotherapy due to their key role in CD8^+^ T cell priming and early CD4^+^ T cell activation, which induce a strong and durable antitumor immunity (45). Unlike macrophages, monocytes and platelets that mediate hepatotoxicity, CRS and thrombocytopenia, respectively, cDC1 activation by CD40 agonist do not contribute to any of these dose-limiting toxicities.

Collectively, these findings suggest that different cellular pathways and locations are engaged by CD40 agonists, which determines the balance between antitumor immunity and side effects. Macrophages and, specifically, liver-resident Kupffer cells are the key population that is engaged by CD40 agonist to mediate hepatotoxicity, in which neutrophils and platelets have also been implicated. Other evidence suggests that IL-6 secretion by monocytes underlies CRS induced by CD40 mAbs.

## Approaches to Increase the Therapeutic Window of CD40 mAbs

### Intratumoral Administration

Understanding the mechanisms driving the antitumor immunity of CD40-targeted immunotherapy, as well as those causing adverse effects, provides a rationale on how to improve the efficacy and safety profile of existing treatments. For example, the finding that the location of immune activation is associated with distinct outcomes, i.e., antitumor activity vs. systemic toxicity, advanced approaches aiming to direct CD40 agonism selectively to the tumor microenvironment (TME) to avoid toxicity. One such strategy is an intratumoral route of mAb administration (30, 46) (**Figure 1**). Indeed, preclinical studies demonstrated a safe profile and lack of hepatotoxicity and thrombocytopenia when anti-human CD40 mAb was administered intratumorally. Treatment resulted in T cell activation and was shown to induce abscopal effects characterized by systemic antitumor T cell activity and long-term memory response (30, 31, 46, 47). Comparison of biodistribution profiles after local or systemic anti-CD40 mAb administration in bladder cancer model revealed that local injection led to CD40 mAb accumulation in the draining lymph node and spleen, presumably because of the high density of CD40^+^ immune cells, whereas systemic injection led to higher Ab concentration in the liver and blood circulation (47). In an early clinical study, intratumoral administration of anti-human CD40 mAb (ADC-1013) into superficial lesions was well tolerated and was accompanied by pharmacodynamic responses (48). Another advantage of local CD40 mAb administration is the avoidance of Ab sink effect by cells with high CD40 expression, mainly circulating B cells.

### Tumor-Associated Antigen-Targeted Bispecific Antibodies

While intratumoral administration is a promising approach for some patients, it is not suitable for all tumors and may be limited to patients with primary or metastatic tumors near the skin, intravesical treatment of bladder cancers, and tumors that are accessible to radiographically directed therapy. This highlights the need to reduce the toxicity of CD40-targeted immunotherapy through systemic administration. One proposed solution is a bispecific antibody (bsAb) that contains a binding arm to tumor-associated antigens (TAA). The rationale behind this approach is that the anti-TAA arm will direct the antibody to the TME and activates APCs locally, thereby avoiding systemic immune stimulation and reducing toxicity **(** **Figure 1** **)**. The first developed CD40 bsAb is ABBV-428, which is constructed from a CD40 arm and a mesothelin TAA (49). This molecule was designed to engage the TME due to the overexpression of mesothelin by several types of tumor cells (50). Indeed, preclinical studies with ABBV-428 suggested less systemic toxicity with similar antitumor immunity compared to the parental monospecific CD40 mAb (49). In a phase 1 clinical trial, ABBV-428 showed a safe profile and the maximum tolerated dose was not reached. However, efficacy was very limited, with no signs of substantial response in patients (51). This outcome could be explained by low expression of mesothelin on tumor cells, that would limit bsAb accessibility to the tumor and thus its ability to crosslink the CD40 receptor, which is required for CD40 signaling (50). Indeed, it was shown in animal models that the expression levels of mesothelin on tumor cell lines dictates the antitumor activity of ABBV-428 (49).

The results of the ABBV-428 trial highlight the need for a bsAb targeting a more highly expressed TAA. 4224 is a CD40/EpCAM bsAb that displayed improved *in vivo* antitumor efficacy compared to the corresponding monospecific CD40 mAb (52). EpCAM is highly expressed on certain tumors and on tumor exosomes, which may induce cross-presentation of tumor-derived neoantigen (i.e., in exosomes or debris), resulting in better priming of tumor neoantigen-specific T cells (52). The toxic profile of 4224 compared to the parental monospecific CD40 mAb has not been reported to date.

A drawback of the CD40/TAA approach is its dependence on sufficient expression levels and density of a specific TAA, which may not be uniformly expressed across different tumor lesions and patients. This may result in variable clinical efficacy, potentially limiting the use of the compound to selective tumor types. In addition, TAA targeted by bsAbs may be required for CD40 crosslinking, similar to FcγRIIB role in neighboring cells. Therefore, their density, membrane fluidity and the binding affinity of the targeting bsAb can substantially affect the potency of these reagents. Because of these limitations, only a handful of tumor surface antigens have thus far been identified as suitable targets for CD40 bispecific antibodies.

### Dendritic Cells-Targeted Bispecific Antibodies

An alternative approach that bypasses the dose-limiting toxicity is to induce CD40 agonism in a cell-specific rather than organ- or tissue-specific manner, by delivering the agonist to the cell population driving treatment efficacy but not toxicity (**Figure 1**). As mentioned, cDC1s mediate the antitumor immunity of CD40 mAb without the toxic side effects. Harnessing this mechanistic understanding, our group recently developed Fc-engineered CD40/DC bsAbs, e.g. CD40/CD11c and CD40/CLEC9A, which exhibit preferred binding and selective activation towards cDC1 populations. This approach improved the therapeutic window of CD40-targeted immunotherapy significantly by increasing antitumor immunity and reducing systemic toxicity *in vivo* in an isogenic mouse model fully humanized for CD40 and FcγRs (44, 53). Importantly, these CD40/DC bsAbs displayed reduced binding and activation of B cells, macrophages and monocytes, the cell types that contribute to sink effect, liver toxicity and CRS. Comparing the mode of action in the TME of CD40/DC bsAb vs its parental monospecific CD40 mAb reveled similar activation of effector CD4^+^ and CD8^+^ T cell response, presumably the result of similar DC engagement by these two types of agonists, leading to DC maturation, expansion and subsequent T cell priming and activation (44). While the monospecific CD40 mAb induces remodeling of the cell state of non-DC CD40^+^ myeloid and B cells in the TME, the DC-targeted bsAb lacks this activity. Despite the more restricted engagement of myeloid cell types in the tumor, the CD40/DC bsAb retains antitumor potency, further supporting the importance of activating the DC-T cell axis for the antitumor activity of CD40 agonists. While this effect was observed in multiple tests in transplantable syngeneic tumor models, further validation in additional tumor types and, eventually, in clinical settings is essential to evaluate the generalization and translational potential of this approach.

**Figure 2:**
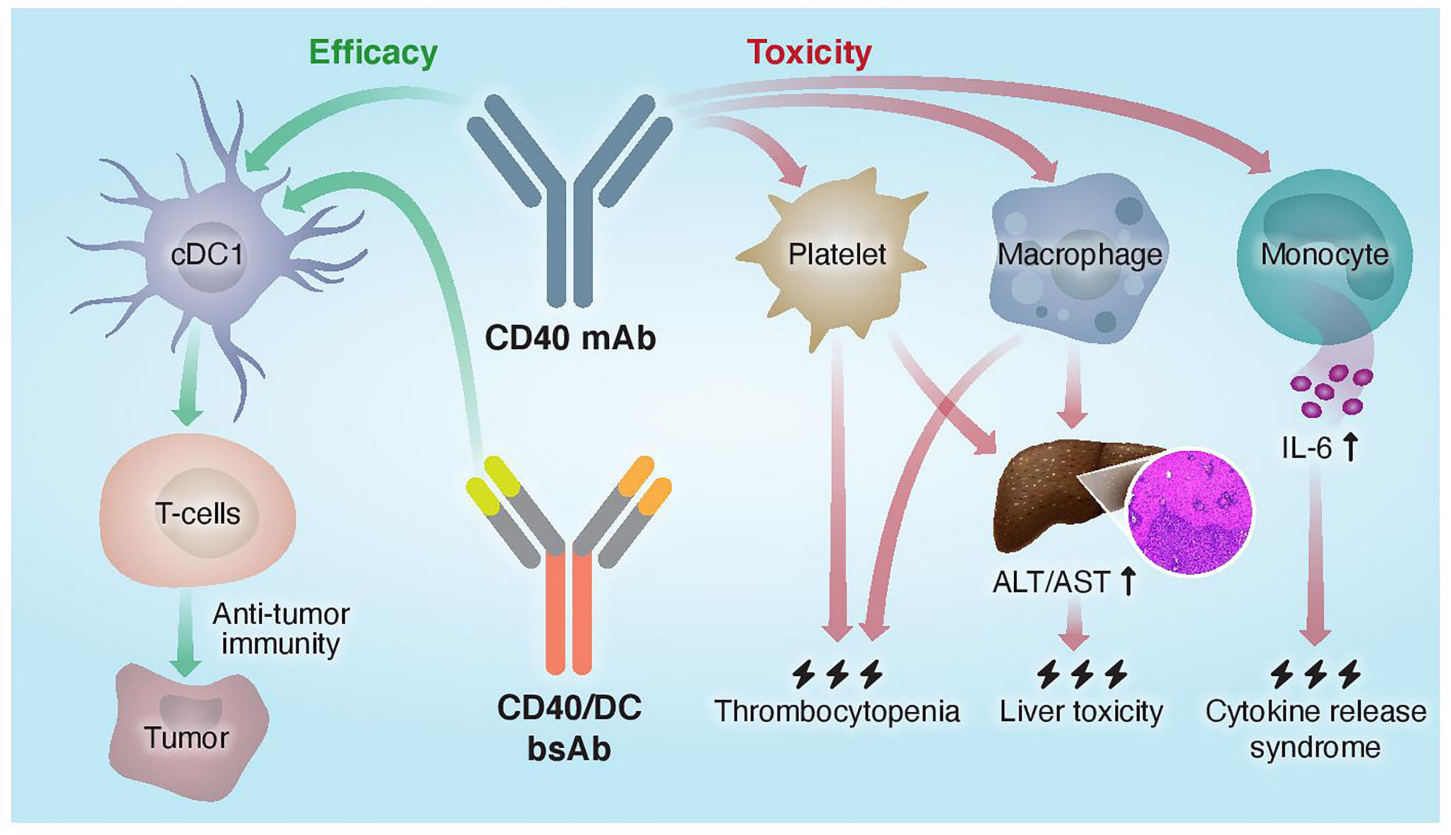
Proposed mechanisms of mono- and bispecific CD40 agonistic antibody activity. Traditional monoclonal anti-CD40 Abs do not distinguish between different CD40^+^ cells and activate both the efficacy arm, driven by cDC1s, and the toxicity arm, driven by macrophages, platelets, and monocytes. CD40/DC bsAbs, which display preferred binding to and selective activation of DC populations, improved the therapeutic window of CD40-targeted immunotherapy by increasing antitumor immunity and reducing systemic toxicity.

The monovalent nature of the CD40 arm in the CD40/DC bispecific format required special considerations in their design. First, these bsAbs exhibit increased sensitivity to FcγR-mediated crosslinking as compared to bivalent IgG formats and Fc engineering was necessary to CD40 clustering and subsequent activation. Second, the monovalent CD40 targeting arm apparently reduces CD40 binding and agonism as compared to a bivalent parental CD40 mAb. However, fine-tuning the affinities of the Fab domains to optimize the DC selectivity of CD40 agonism allows to dose-up these bsAbs without compromising their safety profile, unlike with traditional CD40 mAbs. Thus, this new tri-functional antibody format requires efficient binding to FcγRIIB, CD40, and a DC marker to result in better safety profile and superior antitumor response compared to the parental monospecific CD40 mAb (**Figure 2**).

## Discussion

Driven by recent mechanistic insights into the cellular pathways mediating efficacy and toxicity, as well as the latest developments in antibody and protein engineering, the next generation of Fc-engineered and multi-specific CD40 agonistic mAbs are being developed to bypass toxicity and optimize their potency. Multiple ongoing clinical trials evaluating the safety and potency of these new reagents, either as monotherapies or part of combination therapies, will soon reveal the potential of CD40 agonists as the next wave of immunotherapies. Besides CD40 agonists, the clinical translation of other types of agonistic mAbs has been restricted due to side effects associated with the broad expression profile of their targets on hematological and non-hematological cells. Lesson from pre-clinical and clinical studies of next-generation CD40 antibodies, together with mechanistic knowledge of the cellular pathways that mediate efficacy and toxicity, may enable the development of additional cell- and tumor-selective agonists with an improved therapeutic window.

## Data Availability Statement

The original contributions presented in the study are included in the article/supplementary material. Further inquiries can be directed to the corresponding author.

## Author Contributions

All authors listed have made a substantial, direct, and intellectual contribution to the work and approved it for publication.

## Conflict of Interest

RD is inventor in patents covering Fc-engineered CD40 mAbs. RS and RD are inventors in a PCT patent application covering CD40 bispecific antibodies discussed in this article.

## Publisher’s Note

All claims expressed in this article are solely those of the authors and do not necessarily represent those of their affiliated organizations, or those of the publisher, the editors and the reviewers. Any product that may be evaluated in this article, or claim that may be made by its manufacturer, is not guaranteed or endorsed by the publisher.
